# Ethics of Deep Brain Stimulation in Adolescent Patients with Refractory Tourette Syndrome: a Systematic Review and Two Case Discussions

**DOI:** 10.1007/s12152-018-9359-6

**Published:** 2018-03-23

**Authors:** Anouk Y. J. M. Smeets, A. A. Duits, D. Horstkötter, C. Verdellen, G. de Wert, Y. Temel, L. Ackermans, A. F. G. Leentjens

**Affiliations:** 1 Department of Neurosurgery, Maastricht University Medical Centre, Maastricht, the Netherlands; 2 Department of Psychiatry and Psychology, Maastricht University Medical Centre, Maastricht, the Netherlands; 3 Department of Health Ethics and Society, School of Mental Health and Neuroscience, Maastricht University, Maastricht, the Netherlands; 4 Parnassia Group, PsyQ Nijmegen, Nijmegen, the Netherlands; 5 TicXperts, Heteren, the Netherlands; 6 Department of Health Ethics and Society, Caphri Care and Public Health Research Institute, Maastricht University, Maastricht, the Netherlands

**Keywords:** Tourette syndrome, Tic disorder, Deep brain stimulation, Adolescents, Ethics

## Abstract

**Introduction:**

Tourette Syndrome (TS) is a childhood onset disorder characterized by vocal and motor tics and often remits spontaneously during adolescence. For treatment refractory patients, Deep Brain Stimulation (DBS) may be considered.

**Methods and Results:**

We discuss ethical problems encountered in two adolescent TS patients treated with DBS and systematically review the literature on the topic. Following surgery one patient experienced side effects without sufficient therapeutic effects and the stimulator was turned off. After a second series of behavioural treatment, he experienced a tic reduction of more than 50%. The second patient went through a period of behavioural disturbances that interfered with optimal programming, but eventually experienced a 70% tic reduction. Sixteen DBS surgeries in adolescent TS patients have been reported, none of which pays attention to ethical aspects.

**Discussion:**

Specific ethical issues arise in adolescent TS patients undergoing DBS relating both to clinical practice as well as to research. Attention should be paid to selecting patients fairly, thorough examination and weighing of risks and benefits, protecting the health of children and adolescents receiving DBS, special issues concerning patient’s autonomy, and the normative impact of quality of life. In research, registration of all TS cases in a central database covering a range of standardized information will facilitate further development of DBS for this indication.

**Conclusion:**

Clinical practice should be accompanied by ongoing ethical reflection, preferably covering not only theoretical thought but providing also insights in the views and perspectives of those concerned, that is patients, family members and professionals.

## Introduction

Tourette Syndrome (TS) is a childhood onset neuropsychiatric disorder with multiple motor tics and one or more vocal tics, lasting longer than 1 year. It affects approximately 0.3–1.0% of the general population [1–3]. The majority of patients suffer from associated comorbid conditions such as Attention Deficit and Hyperactivity Disorder (ADHD), obsessive compulsive behaviour (OCB), anxiety, and affective disorders [4]. Typically, tics start around the age of 4–6 years, multiply and worsen until the age of 12–14 years and are then followed by a steady decline in severity. TS is often self-limiting, with symptoms remitting around the late adolescence. However, in about 20% of patients, tics continue in adult life and require chronic treatment [1, 3]. Despite behavioural therapy or pharmacological treatment, a small subgroup of patients fails to respond to treatment and continue to experience significant symptom burden throughout life [5–7]. For those patients, Deep Brain Stimulation (DBS) surgery can be an effective last resort treatment [8]. It must be realized however, that TS is not (yet) a recognized indication for DBS treatment by the Food and Drug Administration (FDA) and the European Commission (EC). We have a longstanding experience with DBS surgery in TS patients since in our centre we performed 16 surgeries in the past 18 years, which is 10% of all reported cases worldwide [9–13].

The emergence of psychiatric disorders, such as depressive disorder, Alzheimer’s disease and anorexia nervosa, as potential indications for DBS as well as the application of DBS in younger patients has created new interest in ethical questions and social challenges [14, 15]. For most of these indications, ethical considerations have been published [16–18]. To date, no ethical considerations have been published on DBS in TS patients, let alone for younger TS patients. The minimum age for considering DBS in TS patients has been widely debated. In 2006, the first guidelines of the Tourette Syndrome Association (TSA) [19] proposed a minimum age of 25 years to ensure that individuals who might experience spontaneous tic remission would not be implanted with a surgical device. More recently, compelling arguments have been made for consideration of surgical intervention at younger ages in certain cases of severe TS. Especially during adolescence, the impact of severe and refractory tics on daily functioning is large. Patients are often not able to go to school or work, they tend to avoid social activities, are not able to develop friendships or an affective relationship, and have a low self-esteem. This developmental interference may have long-lasting effects in later life. Another reason to consider DBS earlier in life is the risk of self-injurious tics e.g., violent cervical tics which may cause cervical myelopathy and secondary neurological deficits [20–22]. This form is sometimes called ‘malignant’ TS and occurs in about 5% of patients [23]. Delaying or not performing DBS in these patients could eventually lead to irreparable harm. Therefore, in more recent TSA recommendations [24] the age guideline has been adjusted and they recommend a multidisciplinary evaluation and discussion, without setting a strict minimum age.

This paper deals with ethical issues associated with DBS of adolescent TS patients. Adolescence is the period of transition from childhood to adulthood. The World Health Organization (WHO) defines adolescence as the age period between 10 and 19 years [25, 26], however the period of adolescence has often been extended to include the ages of 10 through 22–25 years, with most researchers dividing this age span into early (10–13), middle (14–17) and late (18 - mid-20s) adolescence [27]. Therefore, we define adolescence as the age period between 10 and 25 years in this paper. We describe our experiences with the treatment of two adolescent TS patients, both 19 years old, and systematically review the scarce literature on this subject. We discuss the ethical issues and specific difficulties that physicians may encounter when treating adolescent patients.

## Methods and Results

### Patient 1

Patient 1 is a 19-year-old male who experienced tics since the age of five. No other diseases or comorbidities were present, and he had a negative family history for tic syndromes. The tics started with sniffing sounds and tapping his chest, but gradually expanded and increased over the years. At the age of 18, vocal tics, such as almost continuous and loud barking and coprolalia, were the most debilitating. He also suffered from frequent motor tics such as eye blinking and head shaking. Because of the disruptive nature of the tics, he had to leave school and avoided social activities. Different pharmacological treatments as well as behavioural therapy had not been effective in the past. From 2005 till 2013 he used clonidine, risperidone, pimozide and aripiprazole, either solely or in combination, without a positive effect on tics and/or with severe side effects. Moreover, botulin injections did not result in tic improvement. In 2010, he received cognitive behavioural therapy for several months without any improvement. He fulfilled all our criteria for DBS surgery (Table 1), which are in line with the TSA guidelines for DBS in TS [24]. After a multidisciplinary evaluation, he was indicated for bilateral DBS of the anterior internal globus pallidus (GPi) and the surgery was carried out successfully in March 2014 (at the age of 19). Details about the neurosurgical procedure have been published previously [10, 11]. Preoperative he scored 39 (motor/vocal = 14/25) on the Yale Global Tic Severity Scale (YGTSS) [30].

**Table 1:** Inclusion and exclusion criteria for DBS surgery

Inclusion criteria *	Exclusion criteria *
- Primary diagnosis of TS according to the DSM-V criteria [28] - A minimum score of 80 on the Diagnostic Confidence Index [29] - A minimum score of 25 on the Yale Global Tic Severity Scale [27] - Failure to respond to, or intolerable side effects of three-months trials of adequately dosed classical (e.g. haloperidol) and atypical (e.g. risperidone, olanzapine, quetiapine) antipsychotic medication or clonidine - Completed at least ten sessions of behavioural therapy (e.g. exposure and response prevention or habit reversal) - A stable psychosocial environment - Neuropsychological profile indicates candidate can tolerate demands of surgery, postoperative follow-up, and possibility of poor outcome - Age is not a strict criterion. Local ethics committee involvement for cases involving persons <18 years, and for cases considered “urgent” (e.g. impending paralysis from head-snapping tics)	- Tics not related to TS - Major psychiatric disorders (e.g. schizophrenia or bipolar disorder) - Current substance abuse or dependence (except for nicotine) - Severe cognitive impairment - Structural abnormalities on brain magnetic resonance imaging - General contraindications for surgery or anaesthesia

*DBS* Deep Brain Stimulation, *TS* Tourette Syndrome, *DSM-V* Diagnostic and Statistical Manual of Mental Disorders criteria (fifth edition)

*These criteria are in line with the latest worldwide recommendations for DBS in Tourette Syndrome [24]

A few days after surgery, the stimulator was turned on and different contact points and stimulation parameters were tested. With increasing voltages, he experienced side effects such as hyperkinesia, dyskinesia in the legs and a dejected mood, complicating the programming. Therefore, the voltage was reduced before he was discharged, accepting a suboptimal tic reduction at that moment. The expectation was that these side effects would diminish due to habituation and that the voltage could be increased slowly at the outpatient department. However, after 6 months of intensive outpatient visits and a second admission at the hospital to systematically check all possible stimulation parameters, he still experienced the same side effects at all contacts without sufficient therapeutic effects. We decided to turn the stimulator off. At that time, his score on the YGTSS was 24 (motor/vocal = 10/14), which was a relevant reduction compared to the pre-operative score. However, he still suffered from severe tics, e.g. constantly loud barking and headshaking, he felt ashamed and avoided social activities. In September 2016, at the age of 21, he started behaviour therapy again for 3 months with a further relevant improvement in tics, self-confidence and overall functioning. His YGTSS further declined to 18 (motor/vocal = 8/10), 2 years after surgery. In a last outpatient appointment, he was satisfied with his current disease severity and stated that with this symptom severity he would in retrospect not have considered DBS. At this moment, he does not want the stimulator removed since he experiences no burdens and does not want to undergo surgery again.

### Patient 2

Patient 2 is a 19-year-old male adolescent that developed his first motor tics at the age of eight. No other comorbidities were present, and he had a negative family history for tic disorders. Tics started with head shaking, followed by eye blinking and grimaces, and later progressed to his shoulders, arms, trunk and legs. The most debilitating tics were persistent bouts of motor tics in his entire body. He also experienced mild vocal tics like coughing, sniffing and grumbling. At the age of 18, he had to quit school since he was not able to concentrate anymore due to the severity of the tics and because his tics were an unacceptable distraction to fellow students. Medication and behavioural therapy had not been effective in the past. He had used clonidine, pimozide, haloperidol and quetiapine either without lasting positive effects or with intolerable side effects. Between 2007 and 2009 he underwent several psychotherapeutic therapies, among which exposure and response prevention, habit reversal and relaxation training. He was using cannabis daily since he felt it calmed him. This patient fulfilled our selection criteria (Table 1) and was indicated for bilateral DBS of the anterior GPi. He agreed to stop using cannabis as part of the treatment agreement, so that better evaluation of symptoms during programming would become possible. The surgery was carried out without complications in March 2016 (at the age of 19).

Within 1 week after the surgery the stimulator was turned on and systematically different stimulation parameters were tested. Initially he experienced increased agitation, but also an increase in tic frequency and severity. Despite he was informed that finding the correct stimulation parameters would take time, he had hoped for an immediate effect on his tics and was disappointed. This resulted in tension, anger and arguments with his supporting parents, making the programming sessions more difficult. In the following weeks, we had several programming sessions at the outpatient department, mainly decreasing the voltage and slowly increasing it at home, and finally with a voltage of 3.0 a significant tic reduction was established and the agitated feeling had almost disappeared. However, at home the situation escalated, mainly due to the daily excessive use of cannabis, with increasingly quarrelsome and aggressive behaviour. He still lived at home with his parents and siblings who tried to support him and this lead to severe conflicts. The exact reason why he started using drugs again is uncertain, but he told us he was overwhelmed, felt a lot of pressure to succeed with the therapy, and needed the cannabis to calm down, even though he knew it caused him more problems. We offered psychological treatment, which he rejected.

In July 2016, we arranged a meeting with the Committee on Medical Ethical Issues of our hospital to discuss how to proceed: we were not able to reliably evaluate the effects of the DBS due to the excessive use of cannabis and non-compliance of the patient and doubted whether continued treatment was useful. Also, the potential contribution of the DBS to the aggressive behaviour was discussed. He was known with aggressive behaviour following cannabis use and it seemed unlikely that the DBS settings were the cause of his behavioural problems. Therefore, we decided to continue treatment because of its positive effect on tics. However, we discussed with the patient that we would not further adjust the stimulation settings if he would not be compliant with all aspects of the treatment agreement, including stopping cannabis usage. A few months later, his behaviour drastically changed in a positive way. He stopped smoking cannabis, his aggressive behaviour as well as the relationship with his parents improved, and he found a job and started working again. One year after surgery, he was quite satisfied with the effects of DBS. All tics were still present, but much less frequent and intense and he could suppress them better. He did not suffer from side-effects anymore. At that moment, his YGTSS was reduced from 32 preoperatively (motor/vocal = 23/9) to 10 postoperatively (motor/vocal = 8/2).

### Overview of the Ethical Literature

**Fig. 1:**
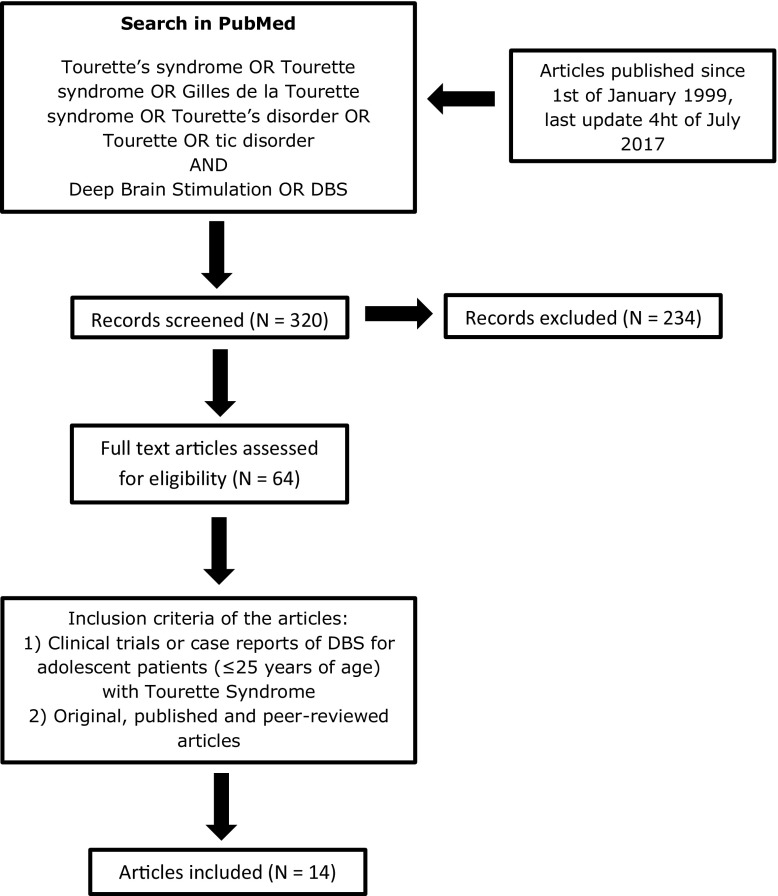
Literature search

We systematically searched the literature for papers discussing ethical issues in young TS patients treated with DBS. We searched PubMed with the search strategy; (Tourette’s syndrome OR Tourette syndrome OR Gilles de la Tourette syndrome OR Tourette’s disorder OR Tourette OR tic disorder) AND (Deep brain stimulation OR DBS) without any time or language restriction (Fig. 1). Since 1999, 156 cases of DBS treatment in TS patients have been published [31]. Of these, only 16 surgeries in patients under 25 years of age have been described in case reports (Table 2) [20–22, 32–42]. These reports mainly focus on target selection, stimulation parameters, and effects on tics and comorbid psychiatric disorders. The most common reasons for operating on these young patients were the severity and intractability of the disease, the considerable influence on daily functioning (e.g. not able to go to school or work, avoiding social activities, not able to develop friendships, and low self-esteem), and severe motor tics causing self-injurious behaviour [20–22, 34–37, 39, 41, 42]. None of these reports discussed the ethical aspects of DBS in adolescent patients. Moreover, minimal attention was paid on to the specific difficulties encountered in these younger patients.

**Table 2:** Overview of the literature about deep brain stimulation in Tourette patients ≤25 years

Author (year)	Number of patients	Age (years)	Sex	Target	Comorbidities	Follow-up (months)	YGTSS (% improvement)	Complications of the surgery	Side effects	Ethical aspects
Shahed [32]	1	16	M	GPi (posterior)	Anxiety, Depression, ADHD	6	84%	None	Not mentioned	Not considered
Dueck [33]	1	16	M	GPi (posterior)	Mental retardation (IQ 60)	12	No change	None	Nausea, dizziness, anxiety (high V)	Not considered
Vernaleken [34]	1	22	M	Thalamus (pf, dmn, lm)	ADHD, OCB, depression	6	36%	Not mentioned	Not mentioned	Not considered
Idris [29]	1	24	M	Thalamus (voi, cm, pf)	None	2	Not performed	Intracerebral hematoma	Not mentioned	Not considered
Kaido [30]	3	19/20/21	1 M, 2F	Thalamus (voi, cm, pf)	OCB/depression in 1 patient	12	44%/31%/29%	None	Blurred vision (high amplitude)	Not considered
Pullen [31]	1	17	M	Thalamus (cm, pf)	ADHD, OCB, borderline	18	82%	None	Not mentioned	Not considered
Hwynn [35]	1	15	M	GPi (posterior)	Dystonia	36	Not performed	None	Not mentioned	Not considered
Savica [23]	2	17/17	2 M	Thalamus (cm, pf)	OCB, ADHD, depression, SIB	12	69%/80%	None	Transient paraesthesia	Self-injurious tics reason for inclusion
Duits [10]	1	20	F	Thalamus (voi, cm, pf)	Depression, autism, OCB, SIB	36	Not performed	None	Hypertonia, mutism, unconsciousness	Severe comorbidity as exclusion criteria
Dong [36]	1	22	M	Unilateral GPi (posterior)	None	12	53%	None	None	Not considered
Massano [37]	1	15	M	GPi (anterior)	OCB, anxiety, depression	24	61%	None	None	Not considered
Huasen [24]	1	19	F	GPi (anterior)	None	12	55%	Not mentioned	Not mentioned	Cervical myelopathy due to tics
Zekaj [38]	1	17	M	Thalamus (voi, cm, pf)	ADHD	72	98% (without DBS)	None	None	Temporary DBS during adolescence
Hauseux [22]	3	12/17/18	3 M	GPi (anterior & posterior), thalamus (cm, pf)	ADHD, OCB, depression, anxiety	47/40/69	18%/6%/32%	None	Dysarthria, worsening OCB/mood	Not considered

*YGTSS* Yale Global Tic Severity Scale, *M* Male, *F* Female, *GPi* globus pallidus internus, *Pf* parafascicular nucleus, *DMN* dorsomedial nucleus, *LM* Lamella Medialis, *Voi* nucleus ventro-oralis internus, *CM* centromedian nucleus, *ADHD* Attention Deficit Hyperactivity Disorder, *IQ* Intelligence Quotient, *OCB* obsessive-compulsive behaviour, *SIB* Self-Injurious Behaviour, *V* Voltage

Most research groups used the minimum age of 25 years as an inclusion criterion for surgery, but some groups did not follow this advice and therefore larger studies might have also included some younger patients [43–52]. In the latest TSA guidelines, 33 patients were reported that had DBS before the age of 25 years. The risk of surgical complications and adverse events did not appear to be higher in this age group compared with older patients [24]. Servello et al. [44] concluded that in their series of 18 patients implanted with thalamic DBS electrodes, three of the four cases who were implanted before the age of 20 years had less satisfactory results after 3 to 6 months than those older than 20 years. This was most likely due to spontaneous waxing and waning of symptoms requiring frequent DBS programming. So far, detailed case descriptions are lacking and no attention has been paid on the ethical issues and specific difficulties that physicians may encounter when treating adolescent TS patients. As such we conclude that there is yet no literature on ethical aspects of performing DBS on TS patients, let alone on adolescent TS patients.

## Discussion

We have described our experiences with DBS in two severely affected and refractory adolescent TS patients. Three years after surgery one patient experienced a tic reduction of more than 50% with the stimulator turned off. The reduction may be due to natural waning of tics that often follows adolescence and due to an additional behavioural treatment for tics after the DBS. At present, he is satisfied with the level of symptoms and would not consider the surgery anymore. The second patient is responding well to the stimulation, but went through a period of behavioural disturbances which are not uncommon during adolescence but interfered with optimal programming. We believe that the experienced stress before, around, and after the surgery might have provoked the excessive use of cannabis and caused the related behavioural disturbances.

DBS in TS seems to have a promising future in terms of tic reduction, however, it raises several ethical questions that need continuing discussion especially in adolescent patients. ***Five major topics*** concerning the ethics of DBS in clinical practice have been identified previously: 1) selecting patients fairly, 2) thorough examination and weighing of risks and benefits, 3) protecting the health of children in paediatric DBS, 4) special issues concerning patient’s autonomy, and 5) the normative impact of quality of life measurements [53].

When discussing the ***first topic***: selecting patients fairly, one of the first questions is whether DBS should be indicated for adolescent patients with TS, given the chances of natural remission during adolescence. In 40% of TS patient’s tics decrease during adolescence, in another 40% they may disappear completely and only in 20% they remain in their full intensity and require chronic treatment [1, 3]. Chronic treatment may include pharmacotherapy and behavioural or psychosocial therapy [6, 7], but there is a small subset of patients who fail to show clinical improvement, and experience intolerable side-effects or potentially life-threatening tics or self-injurious behaviour. These patients are potential candidates for a surgical intervention. The available research shows that DBS of different targets in TS is effective and results in a mean tic improvement of 53% on the YGTSS [31].

Based on the new insights and guidelines we have waived our previous age limit of 25 years [24]. This brings us to incorporating the ***second topic***: thorough examination and weighing of risks and benefits. Our motivation for waiving this age limit was that severe TS in adolescence can be severely disruptive to development, often jeopardizing educational and job opportunities, social interactions and relationships. Delaying surgery in these younger incapacitated TS patients could potentially result in permanent harm to social, psychological, and intellectual development, even if the symptoms eventually subside with age. Similarly, in rare cases of ‘malignant TS’, the tics themselves may carry greater risk for bodily harm or even death [20–22, 24]. An early intervention with positive results may bring significant benefits on the individual’s development and future. On the other hand, DBS is an invasive treatment with potential surgical complications and adverse events. Stimulating the anterior GPi has been related to side-effects such as higher anxiety levels, dyskinesia, fatigue, dizziness and a case of hypomania [31, 54]. Stimulating different targets can cause other side-effects such as gaze disturbances, mood deterioration, apathy and dysarthria [31]. In many instances adjustment of the stimulation parameters can diminish or eradicate these side-effects. Surgical complications mostly centre around hardware malfunction and infections [55, 56]. In general DBS is considered as a safe surgical procedure with a minimal risk (<1%) of serious complications (e.g. bleeding, neurological deficits) [24, 31, 55]. Thorough selection of patients who should be offered DBS is an essential requirement for a successful intervention from a clinical as well as an ethical perspective. In general, patient selection should optimize the individual risk–benefit ratio [53].

Both our patients suffered from severe and refractory TS for many years and were selected for DBS after a thorough multidisciplinary evaluation. Patient 1 had received behavioural treatment and several medications without success and hence he met the DBS inclusion criteria. After unsuccessful DBS, he tried a behavioural treatment again, from which he reported a positive effect on tic severity, self-confidence and quality of life. Behaviour therapy implies doing homework exercises, which this patient reported he had not done the first time he underwent this treatment, because at that time he could not focus on this treatment since he was too busy with school and his parents were in a divorce. Although this patient met the inclusion criteria for DBS, an additional step before starting DBS could have been thorough evaluation of which interventions for tics have been done before and why the patient did not profit from it. After optimizing these relatively first-line less invasive interventions [7] DBS might be an option for further treatment.

A gap in our current state of knowledge is that it is not possible to predict the natural course of tics in TS, so that expected chronicity cannot be used to indicate DBS treatment. At present it remains unclear whether (and to what extent) severely affected patients at the age of 18 may or may not await a significant improvement until the age of 25 [8]. Clinicians selecting DBS candidates would benefit from knowledge of predictive factors, both epidemiological as well as biomarkers, that would allow identification of patients who will remain severely affected into adulthood, but so far this is not possible. Therefore, the advantages and disadvantages of DBS should be evaluated multidisciplinary for every individual adolescent TS patient who may be a surgical candidate. Our patients were informed about the possibility of natural improvement or remission and the possibility of limited or no effect of the surgery on tics. Apparently, they chose to have the operation because of their actual suffering, and not because of the chance of chronicity. In patient 1 as well as in the case described by Zekaj et al. [42], tic severity significantly declined during adolescence, to such an extent that the stimulator in these patients was turned off and they would not be eligible for DBS anymore. Should we not have operated on these patients? With the knowledge of hindsight probably not, but that does not help in the decision making beforehand. On the other hand, if DBS resulted in positive stimulation effects in this very important period of life, would it be ethical to deprive them from this therapeutic option? Moreover, would it be ethical to consider DBS a temporary therapeutic application? And if so, should we remove the stimulator when, after turning off the system, there is no increase in tics for a certain period? It is important to realize that since DBS may be an effective procedure in selected treatment-resistant adolescent TS patients, one may do harm to patients not only by performing DBS, but also by not performing it. It seems to be justified to leave traditional reticence (based on the substantial chance of natural remission) behind and allow earlier intervention in view of the combination of acute suffering and the chance of irreparable harm. The patient’s benefit is central in the ethical evaluation. This criterion can outweigh risks and side-effects, and can make DBS appropriate even in adolescent TS patients with a chance of natural remission [53].

The ***third topic*** is protecting the health of children in paediatric DBS. This can be expanded to protecting the health of adolescent TS patients selected for DBS (age 10–25 years). Local ethics committee involvement is necessary for cases involving persons under 18 years and for cases considered “urgent” in our centre [24]. Moreover, following all the in- and exclusion criteria and basing the decision on a multidisciplinary decision is mandatory. Successful application of the DBS procedure requires much more than the surgery itself. In addition, guidance before, during and after surgery and defining realistic expectations are of major importance. Programming the stimulator to gain the settings with the best effects on tics and no or minor side-effects may take months to a year [24]. This is clearly different from, for example, Parkinson’s disease or essential tremor in which an immediate effect can be observed. Significant spontaneous waxing and waning of symptoms in TS requiring frequent DBS programming makes it even more difficult. The influence of this might be greater during adolescence and spontaneous waning may even be a first sign of spontaneous remission [1, 57]. Therefore, the inclusion criteria for surgery should go much further than being purely clinical criteria and consider certain psychological, social and environmental factors too. To get through all the problems and challenges (e.g. school issues and interpersonal relationships) during the period from childhood to adulthood and handle the related stress, it is very helpful for adolescents not only to have the understanding and support from peers and adults, but also to be able to manage the stressors and hassles of everyday life by themselves. The development of coping strategies may support or inhibit positive adolescent adaptation.

Adolescents who lack adequate coping strategies are more likely to show deviant behavior (e.g. substance use) and use avoiding and distancing coping strategies, whereas adolescents with healthy coping try to solve the problems or seek for social support [58]. Ideally, adolescent candidates for DBS surgery have a stable psychosocial environment and an effective and positive coping style. Patients should be able to tolerate the demands of surgery and the postoperative follow-up, and also the ability to cope with the possibility of a poor outcome. As such it is important for all DBS centres who operate on TS patients with a multidisciplinary approach to include an extensive (neuro)psychological evaluation. Since adolescent patients often still live with their parents, a supporting attitude of parents and other relatives is also of significant importance. Even though formally informed consent lies with the young patient only, especially these young patients can profit from an approach in which both the patient and parents, or other close caregivers, are consulted during the DBS trajectory and involved in treatment and follow-up. In patient 2 the stress experienced before, around and after the surgery might have provoked the excessive use of cannabis and the related behavioural disturbances as a result of an avoiding coping style. Psychological support already prior to surgery can help those patients and parents with a less effective coping style to deal with the stress that can accompany the surgery. Psychological support is also helpful to improve overall compliance and other difficulties that may impede successful DBS.

The ***fourth topic*** concerns the patient’s autonomy and the decision for surgery should be based upon autonomous decision-making of the potential patient [28, 29]. Respect for patient’s autonomy is expressed by safeguarding for informed consent. Some aspects of DBS should be given special attention, namely the complexity of the treatment, the expectations of the patient and his relatives, compliance, and the operating technique [53]. A specific problem in the latter context is the so-called therapeutic misconception, which implies that patients expect evident personal benefit, but fail to realize the innovative character of the treatment and undervaluing the fact that individual treatment response is uncertain and long-term cognitive, emotional and behavioural effects are largely unknown. Desperation is often very high in severely affected TS patients which increases to hope for improvement by DBS. Moreover, depression occurs frequently in adolescent TS patients [59], and their preferences can be more strongly influenced by affective components. Combined, this could lead to the danger of overhasty decisions in favour of undergoing DBS surgery. In addition to informed consent, DBS could have an impact on patient’s autonomy in a separate way. Faced with decision conflicts DBS patients might fail to slow down and make impulsive decisions for their disadvantage [53]. This might have played a role in our second patient. Although he was informed that finding the correct stimulation settings would take time and that we did not expect an immediate effect on his tics, he was very disappointed. This disappointment became part or maybe even the cause of the behavioural problems complicating the treatment even more. Moreover, a stable psychosocial environment, such as supportive parents, is one of the inclusion criteria for DBS and especially important in adolescent patients. Despite being of importance for a positive outcome of DBS, this same environment could also undermine autonomous decision making of the patient. Adolescence is a period of understanding the ‘self’ in relation to the social world with a desire to find a balance [26]. Adolescents can base their decision to take part and undergo treatment on external forms of pressure, either perceived or actual. They may feel ashamed and a burden to their family. These feelings may make them suffer even more than the tics themselves. This may influence their decision to participate and undermines autonomous decision-making and voluntary participation. To address these issues, a good relationship between researcher and patient is essential, given also that life-long treatment and follow-up within the same institution will be needed.

DBS is an elective intervention in TS that is neither life-saving, nor can it cure the disease, and the goal for patients is usually to improve their quality of life, which is greatly limited in refractory TS patients. This brings us to the ***fifth topic***: the normative impact of quality of life measurements. Enquiring for the quality of life of the patients in treatment, additionally to the effectiveness of the intervention, is an important aspect from an ethical point of view. In Parkinson’s disease and other movement disorders, the pure effectiveness of the intervention regarding the motor functions has been considered normatively meaningless [53]. At this point, the question rises whether this also holds in case of TS. In patients with TS the deterioration of their quality of life does not merely consist in the very fact that they do experience these tics, but also in the negative effects the tics have on their surroundings as well as reactions of other people on the tics, resulting in low self-esteem. In addition, DBS treatment in adolescent TS patients has a normative impact on young patient’s long-term quality of life comparable to those described for patients with movement disorders. Delaying the surgery might to lead to deterioration of quality of life during this important period of life. Even though the effects of DBS might be even successful several years after adolescence, irreparable personal and social harm might be done. Tics that are experienced as insulting or disturbing, for example coprolalia or severe motor tics in class room settings, are normatively meaningful because they also affect other persons. Such social consequences and the reactions of others contribute to the fact that young TS patients end up with having their educational and job opportunities ruined and their social interactions and relationships potentially even irreparably harmed. Therefore, the effectiveness of the DBS intervention might be normatively meaningful in itself. The first patient experienced an improvement in tics and quality of life 3 years after surgery, which seemed to be independent of the DBS. The second patient experienced a beneficial effect on his tics which allowed him to start working again and to improve his social contacts, eventually leading to an improvement of his quality of life. At the same time, as discussed before, normatively relevant risks and benefits go further than effectiveness. Too high expectations, disappointment in case there is no immediate success, great suffering pressing patients to seek solutions, troubles with coping with surgery- and treatment-related stress, are all issues that are relevant in this regard.

Although ethical criteria for evaluating psychiatric DBS are principally similar to those discussed for other movement disorders, the experimental character of DBS in TS raises additional questions of research ethics. DBS for TS is not (yet) a recognized indication by regulating authorities and it must be considered a treatment that should only be done in a ***research setting***. This triggers several structural challenges that DBS trials in this domain will unavoidably face. In addition to safeguarding the rights and interests of vulnerable research participants, it would be important to initiate set-up and promotion of high-quality scientific research that could serve the interest of future patients [28]. Today, such trials are not performed worldwide, and such a trial set-up faces several challenges. Even experienced DBS centres perform only a handful of DBS surgeries in TS patients each year, using different techniques and targets, making it difficult to obtain and interpret large-scale outcomes. Multicentre randomised controlled trials would theoretically be desirable, but difficult to realize due to practical reasons and would stretch over many years, and often do not provide conclusive evidence in heterogeneous populations, such as TS patients [52]. Currently the inclusion process, surgical techniques, targets, programming and postoperative care differ largely between different institutions and countries. Moreover, during such study new insights might be gained, e.g. pointing to different surgical targets or other changes in the procedure, which would be unethical to withhold from later participants. Because of these limitations, coupled with the variable effect of surgery, and the overall small numbers of TS patients with DBS worldwide, there is no regulatory agency approval from the FDA and the EC to date. To overcome these structural limitations and to take steps towards larger scale analyses, the TSA recently launched an international Tourette DBS registry and database [60]. The main goal of the project is to share data, uncover best practices, improve outcomes, and provide critical information to regulatory agencies. Such a database can also allow for an analysis of adolescent TS patients to determine for which group of younger patients DBS would be beneficial, either temporarily or lifelong lasting.

## Conclusion

DBS surgery for TS patients has lately been considered at an earlier age because more stress has been placed on the potential long lasting harmful effects of the disorder. Specific ethical issues arise in this age-group relating both to clinical practice as well as to research. In clinical practice attention should be paid to selecting patients fairly, thorough examination and weighing of risks and benefits, including evaluation of adequacy of former applied first-line interventions, protecting the health of children and adolescents receiving DBS, special issues concerning patient’s autonomy, and the normative impact of quality of life. Realistic expectations, consideration of the psychological, social and environmental conditions of adolescent TS patients and the adequate inclusion of relatives in the process of decision-making and care are important. In research, registration of all TS cases in a central database covering a range of standardized information will facilitate the further development of DBS for this indication. Clinical practice should be accompanied by ongoing ethical reflection, preferably covering not only theoretical thought but providing also insights in the views and perspectives of those concerned, that is patients, family members and professionals. Examining the ethical issues of DBS may help to realize its entire potential for benefiting severely suffering TS patients.
